# Gazing at facial features increases dissociation and decreases attractiveness ratings in non-clinical females – A potential explanation for a common ritual in body dysmorphic disorder

**DOI:** 10.1371/journal.pone.0219791

**Published:** 2019-07-25

**Authors:** Anne Möllmann, Antje Hunger, Christina Dusend, Marcel van den Hout, Ulrike Buhlmann

**Affiliations:** 1 Clinical Psychology and Psychotherapy, Institute of Psychology, University of Münster, Münster, Germany; 2 Faculty of Social Sciences and Cultural Studies, University of Applied Sciences Düsseldorf, Düsseldorf, Germany; 3 Faculty of Social and Behavioural Sciences, Utrecht University, Utrecht, The Netherlands; University of São Paulo, BRAZIL

## Abstract

Rituals, such as gazing at faces, are common in body dysmorphic disorder (BDD) and appear in cognitive-behavioral models as a maintaining factor. Rituals are also common in obsessive-compulsive disorder (OCD). In contrast to OCD, the proposed associations between rituals and intrusive thoughts/appearance preoccupation have not been empirically investigated for BDD. We examined if the assumed effect of gazing rituals on attractiveness ratings exists and if it is associated with dissociation. In an experiment, we asked *N* = 65 non-clinical females to focus on the nose of a photographed face at pre- and post-test. In between, participants gazed at the nose of either the same (relevant gazing) or another face (irrelevant gazing). We found increasing dissociation after gazing in both conditions and a differentially stronger decrease of attractiveness ratings in the relevant gazing condition. Our findings support the hypothesized effect of gazing rituals on attractiveness evaluation in cognitive-behavioral models for BDD.

## Introduction

Individuals with body dysmorphic disorder (BDD) are preoccupied with perceived defects or flaws in their appearance, which are mostly related to the face and not or only slightly noticeable to others. Individuals with BDD experience different levels of insight concerning their beliefs about the perceived defects, ranging from good insight to delusional beliefs [1]. Rituals are common in BDD [2,3]. Gazing rituals, such as mirror gazing or gazing at faces in magazines, are one of the most frequent ritual [4]: about 80% of BDD sufferers check their own appearance during mirror gazing or checking rituals, and about 90% compare their own appearance with that of others or gaze at other peoples’ appearances [5].

Cognitive-behavioral models of BDD include gazing rituals as a relevant component [6–8] and a maintaining factor of BDD [3,9,10]. Specifically, BDD models hypothesize that rituals are performed in reaction to distressing feelings, which emerge from beliefs about the unattractiveness of the perceived flaw. Further, the models assume that gazing rituals strengthen these negative beliefs as well as the salience of the perceived flaw [4].

As BDD and OCD share common features, such as intrusive thoughts and rituals [8,11], it is not surprising that BDD models are similar to OCD models [12]. Importantly, models of both disorders propose the same feedback loops: first, rituals reduce distressing feelings and second, these rituals reinforce the salience of relevant triggers and intrusive thoughts as well as the strength of dysfunctional beliefs [4,12]. The content of intrusive thoughts in BDD is highly related to the evaluation of attractiveness, whereas intrusive thoughts in OCD are associated to uncertainty [4].

For OCD, effects of rituals have been empirically investigated for different kinds of rituals. The findings imply that OC rituals follow a paradoxical pattern of increasing uncertainty, although they are performed with the intention to reduce that very same uncertainty. For example, our colleagues [13,14] showed that checking causes memory distrust in mentally healthy individuals as well as in individuals with OCD.

For the purpose of our current study, the findings of van den Hout and colleagues [15] are of particular interest. They investigated OC staring, which reminds of BDD gazing rituals, except for the differing relevant stimuli in OCD and BDD. Specifically, they tested the effect of staring at OC-relevant objects (in this study, a gas stove and a light bulb) on dissociation (e.g., derealization, or altered visual perception) and on uncertainty about one’s own perception in 40 non-clinical individuals. Moreover, they investigated whether staring at an object affects dissociation and perceptual uncertainty specifically for the object that has been stared at (relevant staring) or whether it affects dissociation and uncertainty in general (i.e. also for other objects; irrelevant staring). They observed increased levels of dissociation in both conditions. In addition, staring was followed by a significant increase in perceptual uncertainty. This effect was significantly stronger for the relevant than for the irrelevant staring. Given that the same pattern has been found for OC-like checking rituals [14], the authors concluded that rituals, in general, cause increasing uncertainty, and that dissociation might explain this effect. Dissociation in this context was defined as a state of perceived distorted visual perception or as experiences of derealization. Although BDD models propose analog paradoxical effects of gazing rituals on the perception of flaws and the preoccupation with unattractiveness, these associations have not been empirically investigated, so far. Further, associations between gazing and dissociation and perceptual uncertainty have not yet been investigated with BDD-relevant stimuli.

The results of previous research indicate abnormalities regarding visual perception and appearance evaluation in BDD: based on self-report questionnaires, our colleagues found that individuals with (vs. without) BDD spend significantly more time on looking at others’ specific feature(s) [16]. The authors concluded that the process of comparing, particularly of specific body parts, may contribute to the maintenance of BDD, but the study did not further investigate how gazing at others might maintain BDD. Experimental studies found that individuals with (vs. without) BDD shift their visual attention more often onto and remain longer on regions of concern in both their own and others’ faces, but the studies did not investigate the resulting effects on attractiveness ratings [17–19]. Comparing BDD and OCD with regard to visual processing, study findings indicate differences between the disorders. Specifically, individuals with BDD tend to show more aberrant scan strategies when viewing faces than individuals with OCD, each in relation to mentally healthy individuals. Further, individuals with BDD show stronger impairments in facial emotion recognition tasks than individuals with OCD [20].

Up to now, the study findings on differences in attractiveness ratings between individuals with and without BDD are inconsistent. One study showed that individuals with (vs. without) BDD did not differ in their attractiveness ratings of other people’s faces [21]. In contrast, another research group reported lower attractiveness ratings of others’ faces and bodies in individuals with (vs. without) BDD [22]. Both studies focused on the comparison between groups of individuals (BDD vs. no BDD) and stimuli (faces/bodies of varying attractiveness) but did not investigate gazing as a potential factor for differential ratings of attractiveness.

Two studies with mentally healthy individuals offer first indications for an association between an increasing duration of looking at unfamiliar faces and lower attractiveness ratings [23,24]. In these studies, presentation times ranged from 0.2 to 5s and thus do not seem to be ecologically valid for gazing rituals. Nevertheless, the reported findings are inconsistent with the findings of a group that investigated the effect of gazing (for 3.5 min) in non-clinical females [25]. They compared effects on different stimuli (unfamiliar facial photograph versus the own face in the mirror) and reported increasing attractiveness ratings from pre- to post-gazing for the unfamiliar face photograph but no changes for the own face after mirror gazing. For extreme groups of appearance-dissatisfied vs. -satisfied women (each *n* = 16), they found that appearance-dissatisfied women rated unfamiliar faces significantly more attractive after gazing then pre-gazing, and in contrast rated their own face significantly more unattractive after mirror gazing. In contrast, appearance-satisfied women showed no changes in attractiveness ratings for the unfamiliar face and significantly higher attractiveness self-ratings after mirror gazing [25].

In sum, while there is preliminary empirical evidence that BDD is accompanied by abnormal visual attention, attractiveness ratings do not seem to be generally affected. According to previous study findings, gazing might affect attractiveness ratings. In addition, earlier studies on OCD suggest that dissociation might play a decisive role in explaining the paradoxical effect of OC-like staring on perceptual uncertainty. Empirical evidence if gazing leads to the paradoxical effect on attractiveness ratings, that is hypothesized in cognitive-behavioral models for BDD [4], is still poor and empirical evidence on how this paradoxical effect may be explained is still lacking.

Thus, we aimed to further investigate the assumed paradoxical effect of gazing on attractiveness ratings. With regard to the findings on OC rituals, we additionally examined the effect on dissociation and uncertainty, and we differentiated between relevant and irrelevant gazing. Specifically, we hypothesized that 1) gazing increases dissociation, 2) gazing affects attractiveness ratings and 3) the effect of gazing on attractiveness ratings is significantly stronger when evaluating the face that has been gazed at (relevant gazing) than another face (irrelevant gazing). Further, we explored if gazing at a face affects perceptual uncertainty and if it affects individuals’ confidence about their attractiveness rating.

We therefore extended the experiment of our colleagues [15] in the context of gazing at faces in a non-clinical sample. The induction of symptoms or specific cognitive processes (here: gazing) in non-clinical samples has been suggested as a valuable strategy to investigate their causal status [26–28].

## Methods

### Participants

A total of *N* = 65 nonclinical young female individuals participated in the study (*M*_*age*_ = 22.57, *SD*_*age*_ = 5.09). The sample size was determined to be at least N = 40, equal to the study by van den Hout et al. (2008). Eighty percent of the participants were university students, 16.9% were high school students, and 3.1% were doing voluntary work in the social or environmental sector for one year. The experimental conditions did not differ significantly concerning age, *U* (31, 34) = 551.5, *p* = .746, and years of education, *U* (31, 34) = 571.0, *p* = .406.

### Design and procedure

We used two facial photographs as BDD-relevant stimuli, which were taken from the FACES database [29]. The database does not permit to publish the original pictures. However, the stimuli codes can be received from the authors upon request. With regard to the expected age range of the participants, the photographs were chosen from the FACES age category young (19–31 years). To avoid potential confounding gender effects, we used photographs of female faces and included female participants only. We used photographs of two different faces to control for the possibility of differential stimulus effects.

The experiment consisted of three phases: *pre-assessment* (10 s focusing on the nose plus ratings), *manipulation* (10 min gazing at the same or a different face) and *post-assessment* (10 s focusing on the nose plus ratings). Further, the experiment consisted of three two-staged within- and between-group factors: *time* (pre- and post-test), *gazing* (relevant vs. irrelevant gazing), and *stimulus* (face A vs. face B).

The study protocol was approved by the review board of the Institute of Psychology, University of Münster (approval number: 2015-50-AM; date:11/02/2015). Participants were recruited via bulletin boards in the university. Each participant provided written informed consent after the study procedure had been fully explained. The experiment took place under constant light and sound conditions. Participants were randomly allocated to one of the groups using a random number generator. They were seated in front of a screen and were asked to look at the presented photograph for 10 s in each pre- and post-test (phase 1 and phase 3, followed by the ratings), and for 10 min in between (phase 2: gazing). To imitate the gazing habits of individuals with BDD, participants were instructed to focus on the nose(s) of the presented face(s). Stimulus size was 7 by 10 cm at a viewing distance of 80 cm, to keep a visual angle of approximately 5° by 7°. After finishing the experiment, participants were debriefed about the purpose of the study and reimbursed for their participation.

### Assessments

*Dissociation* was measured with five self-report items on visual perception from the clinician-administered dissociation state scale, CADSS [30]. The items were rated on a 5-point-scale from 0 (“not at all”) to 4 (“extremely”), with higher values indicating stronger dissociation. The items were: (1) “The face seemed unreal or dreamlike.”; (2) “It seemed as though the face looked different than I expected.”; (3) “I felt that the colors and intensity of the face had decreased.”; (4) “I perceived the face as if I was in a tunnel, or as if I was looking through a lens.”; (5) “It seemed as though I was looking at the face through fog, as if it was further away and unclear.” The principle component analyses (PCA) with oblique rotation (oblimin) on the five items on dissociation (pre-test) suggested a one-factor solution. The factor with an eigenvalue of 2.78 explained 55.57% of the variance. Internal consistency was acceptable (*Cronbach’s α* = 0.78). We thus calculated the total score of the five items for the analyses.

For the *attractiveness ratings*, we generated two items to assess how participants rated the attractiveness of the presented faces and noses respectively, and one item to assess the confidence about their judgment. Potential differences in aesthetic judgements, when the rated stimulus does not change in objective manners, and in the confidence about those judge-ments might help to understand changing levels of insight in clinical BDD. All items were rated on a 100 mm Visual Analogue Scale (VAS; 0 “not attractive at all”– 100 “very attractive”; 0 “not certain at all”– 100 “very certain”). We calculated a mean score for the first two items and kept the third item about the confidence of the rating separate. Internal consistency of the attractiveness rating was acceptable (*Cronbach’s α* = 0.74).

*Perceptual uncertainty* was assessed with five items on a 100 mm VAS, derived from studies of our colleagues [31–33]. The items, rated from 0 (“does not apply to me at all”) to 100 (“absolutely applies to me”), with higher values indicating higher uncertainty, were: (1) “It was as though I saw it, but it wasn’t definite enough.”; (2) “I saw it in a way, but it was all fuzzy.”; (3) “I realized that I saw it, but the image was not clear somehow.”; (4) “What I have seen during the last 10 s of observing the face, felt reliable.” (reversed); (5) “I felt confident about what I saw during the last 10 s of looking at the face.” (reversed). As the PCA suggested a two-factor solution, we named the two factors *clarity of perception* (*Cronbach’s α* = 0.85) and *reliability of perception* (*Cronbach’s α* = 0.87) and calculated separate mean scores for both factors.

To measure compliance, at the end of the experiment we asked the participants to rate how well they managed to focus on the nose during gazing. The item was rated on a 5-point-scale from 0 (“not at all”) to 4 (“extremely”).

### Statistical analyses

The data were analyzed using SPSS for Windows, Version 24.0 [34] and JASP [35]. We conducted PCAs to investigate the factor structure of the dependent variables after winsorizing outliers (> *M* + 3*SD*) per item. Further, we conducted *t*-tests to test for potential stimulus effects at pre-assessment (face A vs. face B). As there were no pre-assessment differences on any dependent variable (*dissociation*: *t* (63) = 0.01, *p* = .99; *attractiveness rating*: *t* (63) = 0.21, *p* = .84; *confidence*: *t* (63) = -0.59, *p* = .56; *clarity of perception*: *t* (63) = -0.39, *p* = .70; *reliability of perception*: *t* (63) = -0.07, *p* = .94), we did not differentiate between the two faces in the main analyses (sensitivity analyses on all dependent variables are visualized in S1 Fig). The hypotheses were thus tested using 2 x 2 analyses of variance (ANOVA) with the between-person factor *gazing* (i.e. conditions relevant gazing, n = 31, and irrelevant gazing, n = 34) and the within-person factor *time* (pre- vs. post-assessment). Besides traditional null hypothesis statistical testing (NHST), the data were also examined by conducting Bayesian ANOVA, which allows for the computation of Bayes Factors (*BFs*) [36]. Bayes Factors enable us to quantify the evidence for or against the alternative hypotheses, whereas *p*-values from NHST mainly allow an evaluation of the null hypothesis [37]. In other words, a BF compares both hypotheses and estimates, if one of both seems more likely, given the data. The BFs for the interaction terms were calculated as proportion *BF* (main effects + interaction effects) / *BF* (main effects). *BFs* were interpreted as suggested by [38]: classifying BFs into 11 different groups, e.g. *decisive evidence for H*_*1*_ for BF_10_ > 100 or *decisive evidence for H*_*0*_ for BF_10_ < 1/100.

## Results

### Compliance

A one-way ANOVA revealed no significant differences in the compliance item between groups, *F* (3, 61) = 1.52, *p* = .22, indicating that all groups managed equally well to focus on the nose during the experiment. Means and standard deviations for the compliance item indicated that on average participants managed *moderately to well* to focus on the nose, with all *Ms* ≥ *2*.*24* and all *SDs* ≤ 0.81.

### Does gazing increase dissociation?

Means and standard deviations of the 2 x 2 mixed design ANOVA (time x gazing) are presented in Table 1.

**Table 1:** Dissociation, perceptual uncertainty and attractiveness rating.

Measure/ condition	Dissociation (5 items)		Clarity of perception (3 items)		Reliability of perception (2 items)		Attractiveness rating (2 items)		Confidence rating (1 item)	
	Pre	Post	Pre	Post	Pre	Post	Pre	Post	Pre	Post
	*M (SD)*	*M (SD)*	*M (SD)*	*M (SD)*	*M (SD)*	*M (SD)*	*M (SD)*	*M (SD)*	*M (SD)*	*M (SD)*
Relevant gazing *(n =* 31*)*	4.32 (3.37)	7.94* (5.13)	39.61 (26.94)	47.46 (27.89)	39.08 (22.27)	46.05 (24.45)	59.10 (15.40)	41.82*^a^ (20.73)	63.23 (26.61)	60.26 (24.39)
Irrelevant gazing *(n =* 34*)*	4.85 (3.69)	6.32* (4.53)	42.33 (21.93)	39.44 (25.97)	33.90 (20.88)	36.81 (24.68)	54.91 (18.70)	47.75*^a^ (18.87)	66.62 (23.32)	64.94 (26.71)

*Note*. Relevant gazing = AAA and BBB; irrelevant gazing: ABA and BAB; Dissociation = five items of the CADSS (clinician-administered dissociation state scale); Clarity and reliability of perception = items taken from van den Hout et al. (2008); Attractiveness ratings = three self-generated items;

* = significant differences from pre to post with *p* < .001

a. significant interaction time x condition with *p* = .02

Importantly, there was a significant main effect for time, *F* (1, 63) = 15.09, *p* < .001, *η*_*p*_^*2*^ = .19, reflecting that across both conditions, participants experienced higher dissociation post-gazing compared to pre-gazing. The analysis did neither reveal a significant main effect for gazing (relevant vs. irrelevant condition), *F* (1, 63) = 0.43, *p* = .51, *η*_*p*_^*2*^ = .01, nor a significant interaction time x gazing, *F* (1, 63) = 2.68, *p* = .11, *η*_*p*_^*2*^ = .04.

Supporting the results of NHST, the estimated *BF* (alternative/null) suggested that the data were 114.24 times more likely to occur in a model including a main effect for time, rather than in a model without this effect. The *BF* for the additional evidence for the interaction time x gazing was *BF*_*10*_ = 0.81 (*anecdotal* evidence for H_1_ for *BF*_*10*_ [1/3;1]), see Table 2. This *BF* neither speaks in favor of the null hypothesis nor the alternative hypothesis, indicating that, given our data, it remains inconclusive if the interaction effect time x gazing exists.

**Table 2:** Bayesian repeated measures ANOVA of dissociation.

Models	*P(M)*	*P(M|data)*	*BF*_*M*_	*BF*_*10*_	*Error %*
Null Model (incl. subject)	0.200	0.006	0.023	1.000	
Gazing	0.200	0.002	0.006	0.268	1.032
Time	0.200	0.649	7.384	114.240	0.724
Gazing + Time	0.200	0.190	0.936	33.405	2.376
Gazing + Time + Gazing * Time	0.200	0.155	0.731	27.214	3.252

*Note*. All models include subject. *P(M)* = prior model probabilities (before considering the data), *P(M/Data)* = posterior model probabilities (considering the data), *BF*_*M*_ = Bayes factor for the model, *BF*_*10*_ = Bayes factor for this compared to the null model.

### Does gazing influence attractiveness ratings?

Means and standard deviations of the 2 x 2 mixed design ANOVA (time x gazing) are presented in Table 1.

Looking at the attractiveness rating, there was a significant main effect for time, *F* (1, 63) = 32.50, *p* < .001, *η*_*p*_^*2*^ = .34, and a significant interaction time x gazing, *F* (1, 63) = 5.57, *p* = .02, *η*_*p*_^*2*^ = .08, but no main effect for gazing, *F* (1, 63) = 0.05, *p* = .83, *η*_*p*_^*2*^ < .01. Specifically, all participants rated the presented faces as less attractive post-gazing compared to pre-gazing, but the ratings were significantly lower in the relevant gazing condition. The *BF* (see Table 3) for the additional evidence for the interaction time x gazing only was *BF*_*10*_ = 0.94 (*anecdotal* evidence for H_1_ for *BF*_*10*_ [1/3;1]), which speaks neither in favor of the null nor the alternative hypothesis. This indicates that, given our data, it remains inconclusive if the interaction effect (i.e. the experimental manipulation affected participant’s attractiveness ratings) exists.

**Table 3:** Bayesian repeated measures ANOVA of uncertainty of perception (two factors) and attractiveness rating (two factors).

Models	*P(M)*	*P(M|data)*	*BF*_*M*_	*BF*_*10*_	*Error %*	*P(M)*	*P(M|data)*	*BF*_*M*_	*BF*_*10*_	*Error %*
	clarity of perception					reliability of perception				
Null Model (incl. subject)	0.200	0.640	7.096	1.000		0.200	0.386	2.513	1.000	
Gazing	0.200	0.168	0.808	0.263	1.029	0.200	0.256	1.374	0.663	0.898
Time	0.200	0.137	0.632	0.213	1.221	0.200	0.190	0.938	0.492	1.221
Gazing + Time	0.200	0.037	0.152	0.057	3.715	0.200	0.130	0.597	0.337	2.866
Gazing + Time + Gazing * Time	0.200	0.019	0.078	0.030	1.791	0.200	0.039	0.161	0.100	4.374
	attractiveness rating					confidence of the attractiveness rating				
Null Model (incl. subject)	0.200	0.001	0.005	1.000		0.200	0.591	5.775	1.000	
Gazing	0.200	3.892e^-4^	0.002	0.320	1.122	0.200	0.211	1.071	0.358	0.916
Time	0.200	0.588	5.699	483.632	1.711	0.200	0.137	0.634	0.232	1.478
Gazing + Time	0.200	0.212	1.073	174.151	4.662	0.200	0.048	0.203	0.082	1.382
Gazing + Time + Gazing * Time	0.200	0.199	0.995	163.998	1.891	0.200	0.013	0.052	0.022	2.911

*Note*. All models include subject. P(M) = prior model probabilities (before considering the data), P(M/Data) = posterior model probabilities (considering the data), *BF*_*M*_ = Bayes factor for the model, *BF*_*10*_ = Bayes factor for this compared to the null model.

In contrast, participants, irrespective of gazing condition, did not differ in their confidence of the attractiveness rating, reflected by the absence of significant main effects for time, *F* (1, 63) = 0.50, *p* = .48, *η*_*p*_^*2*^ = .01, and for gazing, *F* (1, 63) = 0.57, *p* = .45, *η*_*p*_^*2*^ = .01, as well as the non-significant interaction time x gazing, *F* (1, 63) = 0.04, *p* = .85, *η*_*p*_^*2*^ < .01. Additionally, the *BFs* (see Table 3) indicated *anecdotal to very strong* evidence for the null hypothesis (H0), i.e. the experimental task does not affect participant’s confidence of the attractiveness rating.

### Does gazing influence uncertainty of perception?

The results of the ANOVAS as well as of the *BFs* (see Table 3) indicated no effect of gazing on individuals’ uncertainty of perception. Means and standard deviations of the 2 x 2 ANOVA are presented in Table 1.

There were no significant main or interaction effects on the two factors [*clarity of perception*: time *F* (1, 63) = 0.36, *p* = .55, *η*_*p*_^*2*^ = .01, gazing *F* (1, 63) = 0.30, *p* = .59, *η*_*p*_^*2*^ = .01, time x gazing *F* (1, 63) = 1.68, *p* = .20, *η*_*p*_^*2*^ = .03; *reliability of perception*: time *F* (1, 63) = 2.19, *p* = .14, *η*_*p*_^*2*^ = .03, gazing *F* (1, 63) = 2.38, *p* = .13, *η*_*p*_^*2*^ = .04, time x gazing *F* (1, 63) = 0.37, *p* = .55, *η*_*p*_^*2*^ = .01].4.

## Discussion

In this experimental study, we investigated effects of a BDD-like gazing ritual in non-clinical females to evaluate potential associations between such rituals and the preoccupation with appearance, as proposed in cognitive-behavioral models for BDD [4]. We examined effects of gazing on dissociation, attractiveness ratings, and uncertainty of perception.

### Gazing increases dissociation

As hypothesized, 10 min gazing led to higher levels of dissociation. We found this increase in dissociation from pre- to post-gazing across both conditions (relevant and irrelevant gazing).

Our finding on dissociation replicates the effect of increasing dissociation after staring at objects [15], although facial stimuli are more complex than object stimuli [39]. Further, our finding is in line with previous research on effects of gazing on dissociation: our colleagues reported dissociative experiences after gazing at a dot or at one’s own face in the mirror [40]. Another researcher also observed dissociative phenomena in individuals gazing at their own face in the mirror [41]. Integrating these results, gazing itself seems to increase dissociation over time, apparently irrespective of the kind of stimulus (object, dot, own face in the mirror, photographed face).

Considering that the *BF* of our findings neither speaks in favor of nor against the interaction effect, it remains unclear, if (and if so, to what extent) changing visual input during gazing influences dissociative experiences. However, as gazing rituals seem to cause dissociation, the question emerges, if this causes further negative consequences, for example with regard to the appearance/attractiveness preoccupation.

### Gazing decreases attractiveness ratings

Gazing was followed by lower attractiveness ratings of the presented face. This effect was even stronger if the rated face was the same than during the 10 min gazing period (relevant gazing). Our results suggest that gazing at a face per se lowers attractiveness ratings. However, gazing at a face most strongly decreases the attractiveness rating of the very same face. This finding might be especially helpful in explaining the severe effects of gazing at oneself, e.g. during mirror gazing, on the self-evaluation of attractiveness.

Remarkably, participants’ confidence in their ratings did not change after gazing, that is participants irrespective of condition felt equally confident in their attractiveness evaluation pre- and post-gazing. This finding suggests that even though the decrease in attractiveness ratings after gazing is not based on any changes in objective matters (they rated the same photograph), the subjective devaluation of the rated face nonetheless feels real and reliable for the individual. In conclusion, individuals may believe in their devaluation of appearance after gazing which is likely due to a misperception (probably based on dissociation).

However, one study revealed contrary effects with higher (rather than lower) attractiveness ratings after 3.5 min gazing at a facial photograph [24]. There was also some preliminary evidence that the effect of gazing on attractiveness evaluation might be associated with appearance satisfaction. These inconsistent findings across studies (if and how gazing affects attractiveness evaluation) leave room for future research. We need to further investigate the impact of appearance satisfaction, gazing stimulus qualities (e,g, fixed photographs vs. living pictures in the mirror/ videotape) and the duration of gazing time on attractiveness evaluation.

Our results on attractiveness ratings indicate that gazing might not only, as BDD models assume [4,7], lead to a more critical view on one’s own appearance but also on other people’s appearance. BDD models assume a negative influence of gazing rituals on the preoccupation with one’s own appearance and the perceived flaw, but do not yet address the evaluation of other people’s appearance. Future studies may further investigate this issue and its relevance for the explanation of BDD symptom etiology and/or maintenance.

### Gazing does not influence perceptual uncertainty

We did not find any significant effects on uncertainty of perception nor evidence for those effects as concluded from the corresponding *BFs*. On the one hand, these findings may seem surprising, because van den Hout and colleagues found an increase in perceptual uncertainty (main effect), especially during relevant staring at objects [15]. Thus, they discussed a possible general principle in OC-like experiences, namely that perseveration leads to uncertainty in the related domain (e.g. checking leads to memory uncertainty; staring leads to perceptual uncertainty). Given our current findings for BDD-like gazing at facial features, this does not seem to be a general, transdiagnostic principle with respect to uncertainty.

On the other hand, the inconsistent findings in OCD-related staring vs. BDD-related gazing may become consistent if we propose that the core construct ‘uncertainty’ in OC-related rituals corresponds to the ‘evaluation of attractiveness’ in BDD-related rituals. Since staring (at objects) in OCD is used to become certain about one’s perception to *prevent* harm, inconclusive perceptions of potentially dangerous objects (e.g. stoves) are most likely to trigger uncertainty and thus fan the fear of making mistakes. In BDD, gazing (at faces) is used to *investigate* attractiveness (other than harm prevention). Inconclusive perceptions of faces may therefore most likely impact attractiveness evaluation and have thus the potential to fan preoccupation with appearance. Based on this hypothesis, study findings may follow the same pattern: Staring rituals and gazing rituals both lead to an increase, rather than a decrease, in preoccupation with pathological fears. This assumption is in line with current BDD models, which do address preoccupation with appearance instead of uncertainty [4,7].

Further, the unaffected confidence in participants’ attractiveness ratings pre- to post-gazing implies that gazing at appearance-related stimuli does not lead to doubts about an *accurate view* of the feature. This finding might correspond to the concept of insight. Individuals with BDD often show a lack of insight, that is they are convinced of their accurate and undistorted view of the perceived flaw or defect [1,42]. Compared to BDD, insight is on average less impaired in individuals with OCD [11]. In correspondence, uncertainty increased after OC-related staring [15], but did not change after BDD-related gazing.

### Gazing, dissociation, and attractiveness ratings

We conclude from our findings of an increase in dissociation after gazing and a decrease of attractiveness ratings, most strongly after relevant gazing, that gazing rituals might have a causal effect on the evaluation of attractiveness. This effect might be explainable by dissociative experiences during gazing.

One could argue that dissociation during gazing rituals might impair the accurate processing of the stimulus, for example because it is perceived “unreal or dreamlike”, and therefore prevents a valid memory representation of the stimulus. An inaccurate memory representation of the stimulus might affect the evaluation of other stimulus-relevant dimensions, for example the evaluation of attractiveness in the context of facial stimuli. In this case, gazing at faces increases perceptual inaccuracy, but unlike OC-staring this inaccuracy does not impact the subjective certainty about one’s perception.

The hypothesized negative effect of gazing and dissociation on the attractiveness evaluation of an observed body part might have important implications for the therapeutic work with individuals with BDD. In this case, one may reduce gazing at others’ as well as at oneself as part of ritual prevention. Provided that this negative effect similarly occurs in gazing at own features, mirror exposure tasks may be conducted in a way that prevents gazing and, thus, dissociation. One treatment manual recommends perceptual retraining as one component in the treatment of BDD [4]. Even though they do not refer to dissociation in the intervention, the instructions help to prevent dissociation: patients are instructed to look at themselves in the mirror in a more holistic way. They are instructed not to focus on their perceived flaws but instead to spend a balanced amount of time looking at various parts of the body.

### Limitations and conclusions

There are possible study limitations that need to be addressed. We focused on BDD-like gazing at others, which is one of the most frequent rituals in BDD [5]. Especially with regard to former findings on gazing and dissociation [40,41], there is little reason to believe findings would have been different if individuals had been asked to gaze at their own face. However, future studies may resolve this issue empirically by investigating relevant and irrelevant gazing at the own face. Further, we asked the participants how well they managed to focus on the nose but we did not control the gazing behavior with, for example, eye-tracking.

We examined a non-clinical sample and did not use stress induction pre-gazing to imitate distressing emotions, which are known to trigger engaging in gazing rituals [4]. Note that in studies on effects of repeated checking, individuals with OCD showed the same paradoxical decrease in memory confidence as healthy individuals [43], suggesting the same may hold true for the paradigm reported here. However, the study design limits the generalizability of the findings regarding effects in individuals with clinical BDD. Again, this is an empirical issue that awaits testing in studies with BDD sufferers.

In conclusion, this is the first study to investigate the experimental effects of gazing on dissociation and the evaluation of attractiveness. Our findings provide empirical support for the proposed associations in cognitive-behavioral models of BDD [4] and offer dissociation as a potential explanation for the decreased attractiveness ratings after gazing. We conclude that dissociation might be a key component of maintaining the vicious cycle during BDD-like as well as OC-like gazing rituals, but that in contrast to OC-related staring, dissociation while gazing in BDD affects the evaluation of attractiveness but does not increase uncertainty of perception (nor confidence in attractiveness evaluation). Although more evidence is needed to understand potential pathological effects on body image, this study may help inform therapists to become aware of the potentially negative effects of dissociation and gazing in treatment interventions.

## Supporting information

S1 Fig Pre-post-comparisons between faces and groups (black dots: Relevant gazing; grey dots: Irrelevant gazing) on the dependent variables dissociation, attractiveness rating, and uncertainty of perception. Higher values indicate a) higher dissociation, b) higher attractiveness ratings, c) lower reliability and clarity of perception, i.e. higher uncertainty of perception. Error bars indicate 95% confidence intervals.
(DOCX)

## References

[1] American Psychiatric Association. Diagnostic and statistical manual of mental disorders. DSM. 5th ed Washington, DC: APA; 2013.

[2] Buhlmann U, Winter A. Perceived ugliness: An update on treatment-relevant aspects of body dysmorphic disorder. Curr Psychiatry Rep. 2011;13(4):283–8. 10.1007/s11920-011-0203-5 21538032

[3] Veale D, Riley S. Mirror, mirror on the wall, who is the ugliest of them all? The psychopathology of mirror gazing in body dysmorphic disorder. Behav Res Ther. 2001;39(12):1381–1393. 11758697 10.1016/s0005-7967(00)00102-9

[4] Wilhelm S, Phillips KA, Steketee G. Cognitive-behavioral therapy for body dysmorphic disorder: A treatment manual. New York: Guilford Press; 2013.

[5] Phillips KA. The broken mirror: Understanding and treating body dysmorphic disorder. Oxford: Oxford University Press; 2005.

[6] Fang A, Wilhelm S. Clinical features, cognitive biases, and treatment of body dysmorphic disorder. Annu Rev Clin Psychol. 2015;11(1):187–212. 25581240 10.1146/annurev-clinpsy-032814-112849

[7] Veale D. Advances in a cognitive behavioural model of body dysmorphic disorder. Body Image. 2004;1(1):113–25. 10.1016/S1740-1445(03)00009-3 18089145

[8] Wilhelm S, Neziroglu F. Cognitive theory of body dysmorphic disorder In: Frost RO, Steketee G, editors. Cognitive approaches to obsessions and compulsions: Theory, assessment, and treatment. Oxford: Pergamon Pr. Inc; 2002 pp. 203–14.

[9] Kollei I, Brunhoeber S, Rauh E, de Zwaan M, Martin A. Body image, emotions and thought control strategies in body dysmorphic disorder compared to eating disorders and healthy controls. J Psychosom Res. 2012;72(4):321–7. 10.1016/j.jpsychores.2011.12.002 22405229

[10] Windheim K, Veale D, Anson M. Mirror gazing in body dysmorphic disorder and healthy controls: Effects of duration of gazing. Behav Res Ther. 2011;49(9):555–64. 10.1016/j.brat.2011.05.003 21726855

[11] Phillips KA. Body Dysmorphic Disorder: Advances in Research and Clinical Practice. New York: Oxford University Press; 2017.

[12] Salkovskis PM. Obsessional-compulsive problems: A cognitive-behavioural analysis. Behav Res Ther. 1985;23(5):571–83. 4051930 10.1016/0005-7967(85)90105-6

[13] Tolin DF, Abramowitz JS, Brigidi BD, Amir N, Street GP, Foa EB. Memory and memory confidence in obsessive–compulsive disorder. Behav Res Ther. 2001;39(8):913–27. 11480832 10.1016/s0005-7967(00)00064-4

[14] van den Hout MA, Kindt M. Repeated checking causes memory distrust. Behav Res Ther. 2003;41(3):301–16. 12600401 10.1016/s0005-7967(02)00012-8

[15] van den Hout MA, Engelhard IM, de Boer C, du Bois A, Dek ECP. Perseverative and compulsive-like staring causes uncertainty about perception. Behav Res Ther. 2008;46(12):1300–4. 10.1016/j.brat.2008.09.002 18951122

[16] Anson M, Veale D, Miles S. Appearance comparison in individuals with body dysmorphic disorder and controls. Body Image. 2015;15:132–40. 10.1016/j.bodyim.2015.08.003 26379252

[17] Greenberg JL, Reuman L, Hartmann AS, Kasarskis I, Wilhelm S. Visual hot spots: An eye tracking study of attention bias in body dysmorphic disorder. J Psychiatr Res. 2014;57:125–32. 10.1016/j.jpsychires.2014.06.015 25005739

[18] Grocholewski A, Kliem S, Heinrichs N. Selective attention to imagined facial ugliness is specific to body dysmorphic disorder. Body Image. 2012;9(2):261–9. 10.1016/j.bodyim.2012.01.002 22325851

[19] Kollei I, Horndasch S, Erim Y, Martin A. Visual selective attention in body dysmorphic disorder, bulimia nervosa and healthy controls. J Psychosom Res. 2017;92:26–33. 10.1016/j.jpsychores.2016.11.008 27998509

[20] Malcolm A, Labuschagne I, Castle D, Terret G, Rendell PG, Rossell SL. The relationship between body dysmorphic disorder and obsessive-compulsive disorder: A systematic review of direct comparative studies. Aust N Z J Psychiatry 2018;52:1030–1049. 10.1177/0004867418799925 30238784

[21] Buhlmann U, Etcoff NL, Wilhelm S. Facial attractiveness ratings and perfectionism in body dysmorphic disorder and obsessive-compulsive disorder. J Anxiety Disord. 2008;22(3):540–7. 10.1016/j.janxdis.2007.05.004 17624717

[22] Moody TD, Shen VW, Hutcheson NL, Henretty JR, Sheen CL, Strober M, et al Appearance evaluation of others’ faces and bodies in anorexia nervosa and body dysmorphic disorder. Int J Eat Disord. 2017;50(2):127–38. 10.1002/eat.22604 27566987 PMC5345932

[23] Rashidi M, Pazhoohi F, Hosseinchari M. Effect of facial stimuli exposure time on evaluation of facial attractiveness. Aust J Psychol. 2012;64(3):164–8.

[24] Willis J, Todorov A. First impressions: Making up your mind after a 100-ms exposure to a face. Psychol Sci. 2006;17(7):592–598. 10.1111/j.1467-9280.2006.01750.x 16866745

[25] Mulkens S, Jansen A. Mirror gazing increases attractiveness in satisfied, but not in dissatisfied women: A model for body dysmorphic disorder? J Behav Ther Exp Psychiatry. 2009;40(2):211–8. 10.1016/j.jbtep.2008.10.001 19013550

[26] Abramovitch A, Cooperman A. The cognitive neuropsychology of obsessive-compulsive disorder: A critical review. J Obsessive-Compuls Relat Disord. 2015;5:24–36.

[27] De Putter LMS, Van Yper L, Koster EHW. Obsessions and compulsions in the lab: A meta-analysis of procedures to induce symptoms of obsessive-compulsive disorder. Clin Psychol Rev. 2017;52:137–47. 10.1016/j.cpr.2017.01.001 28119197

[28] Ehring T, Kleim B, Ehlers A. Combining clinical studies and analogue experiments to investigate cognitive mechanisms in posttraumatic stress disorder. Int J Cogn Ther. 2011;4(2):165–77. 10.1521/ijct.2011.4.2.165 23814633 PMC3695549

[29] Ebner NC, Riediger M, Lindenberger U. FACES—A database of facial expressions in young, middle-aged, and older women and men: Development and validation. Behav Res Methods. 2010;42(1):351–62. 10.3758/BRM.42.1.351 20160315

[30] Bremner JD, Krystal JH, Putnam FW, Southwick SM, Marmar C, Charney DS, et al Measurement of dissociative states with the clinician-administered dissociative states scale (CADSS). J Trauma Stress. 1998;11(1):125–36. 10.1023/A:1024465317902 9479681

[31] van den Hout MA, Kindt M. Phenomenological validity of an OCD-memory model and the remember/know distinction. Behav Res Ther. 2003;41(3):369–78. 12600406 10.1016/s0005-7967(02)00097-9

[32] van den Hout MA, Engelhard IM, Smeets M, Dek ECP, Turksma K, Saric R. Uncertainty about perception and dissociation after compulsive-like staring: Time course of effects. Behav Res Ther. 2009;47(6):535–9. 10.1016/j.brat.2009.03.001 19342006

[33] Hermans D, Engelen U, Grouwels L, Joos E, Lemmens J, Pieters G. Cognitive confidence in obsessive-compulsive disorder: Distrusting perception, attention and memory. Behav Res Ther. 2008;46(1):98–113. 10.1016/j.brat.2007.11.001 18076865

[34] IBM Corp. IBM SPSS Statistics for Windows. Armonk, NY: IBM Corp.; 2017.

[35] JASP Team. JASP. 2016.

[36] Wagenmakers E-J, Love J, Marsman M, Jamil T, Ly A, Verhagen AJ, et al Bayesian inference for psychology. Part II: Example applications with JASP. Psychon Bull Rev. 2017; 10.3758/s13423-017-1323-7 28685272 PMC5862926

[37] Wagenmakers E-J, Marsman M, Jamil T, Ly A, Verhagen J, Love J, et al Bayesian inference for psychology. Part I: Theoretical advantages and practical ramifications. Psychon Bull Rev. 2017; 10.3758/s13423-017-1343-3 28779455 PMC5862936

[38] Wetzels R, Matzke D, Lee MD, Rouder JN, Iverson GJ, Wagenmakers E-J. Statistical evidence in experimental psychology: An empirical comparison using 855 t-tests. Perspect Psychol Sci. 2011 5;6(3):291–8. 10.1177/1745691611406923 26168519

[39] Tanaka JW, Farah MJ. Parts and wholes in face recognition. Q J Exp Psychol. 1993;46(2):225–245. 10.1080/14640749308401045 8316637

[40] Miller PP, Brown TA, DiNardo PA, Barlow DH. The experimental induction of depersonalization and derealization in panic disorder and nonanxious subjects. Behav Res Ther. 1994;32(5):511–9. 8042962 10.1016/0005-7967(94)90138-4

[41] Caputo GB. Strange-face-in-the-mirror illusion. Perception. 2010 7;39(7):1007–8. 10.1068/p6466 20842976

[42] Phillips KA, Pinto A, Hart AS, Coles ME, Eisen JL, Menard W, et al A comparison of insight in body dysmorphic disorder and obsessive–compulsive disorder. J Psychiatr Res. 2012;46(10):1293–9. 10.1016/j.jpsychires.2012.05.016 22819678 PMC3432724

[43] van den Hout MA, van Dis EAM, van Woudenberg C, van de Groep IH. OCD-like checking in the lab: A meta-analysis and improvement of an experimental paradigm. J Obsessive-Compuls Relat Disord. 2017; 10.1016/j.jocrd.2017.11.006

