# Supplementary material for: Gazing at facial features increases dissociation and decreases attractiveness ratings in non-clinical females – A potential explanation for a common ritual in body dysmorphic disorder
Source: PLoS One. 2019 Jul 25;14(7):e0219791. doi: 10.1371/journal.pone.0219791 (PMC6657848; doi:10.1371/journal.pone.0219791)
Supplement: S1 Fig — Higher values indicate a) higher dissociation, b) higher attractiveness ratings, c) lower reliability and clarity of perception, i.e. higher uncertainty of perception. Error bars indicate 95% confidence intervals. (DOCX) [file pone.0219791.s001.docx]

## S1 Figure

*
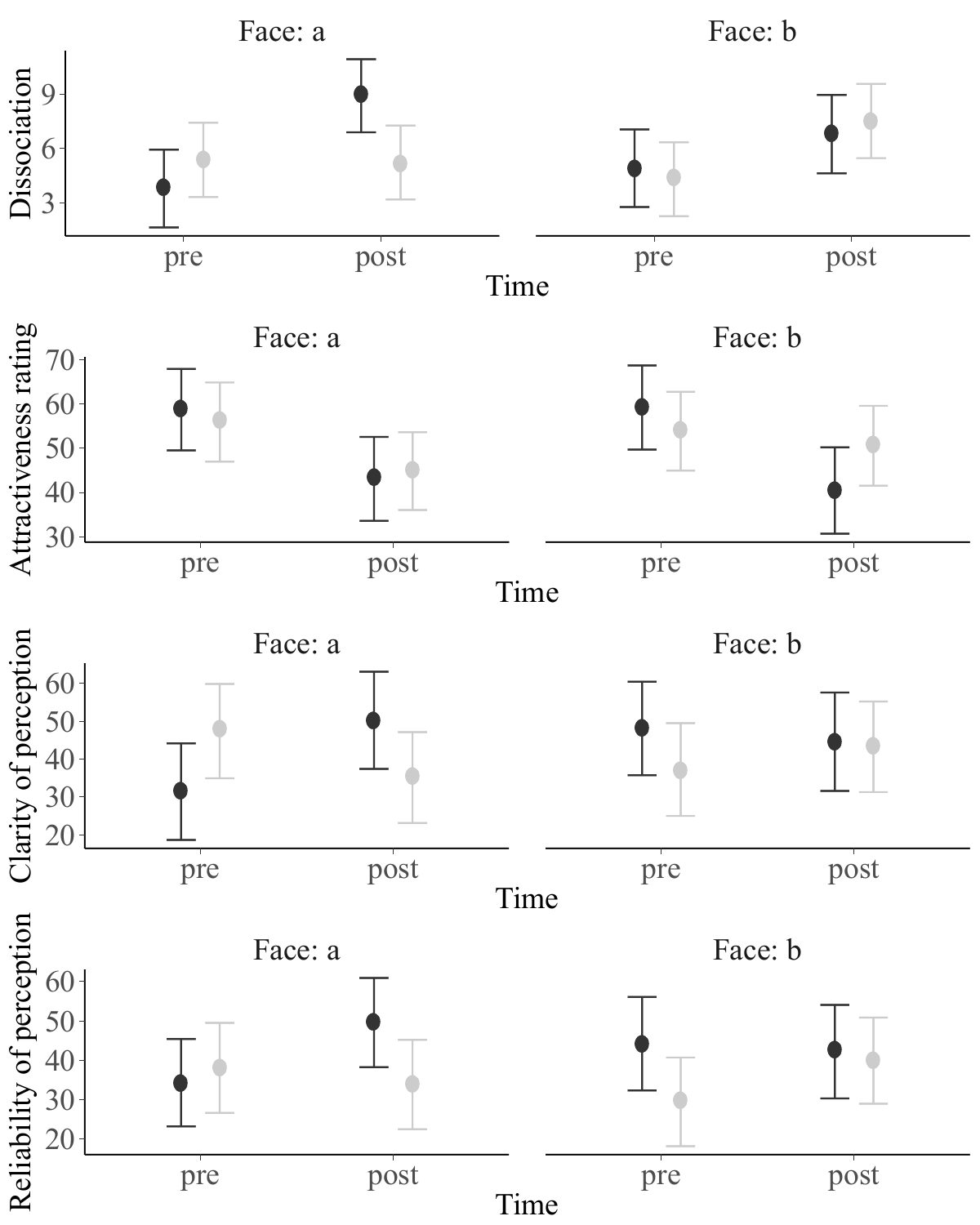
*

**S1 Fig. Pre-post-comparisons between faces and groups (black dots: relevant gazing; grey dots: irrelevant gazing) on the dependent variables dissociation, attractiveness rating, and uncertainty of perception.** Higher values indicate a) higher dissociation, b) higher attractiveness ratings, c) lower reliability and clarity of perception, i.e. higher uncertainty of perception. Error bars indicate 95% confidence intervals.
